# Phenotype of POLE-mutated endometrial cancer

**DOI:** 10.1371/journal.pone.0214318

**Published:** 2019-03-27

**Authors:** Sara Imboden, Denis Nastic, Mehran Ghaderi, Filippa Rydberg, Tilman T. Rau, Michael D. Mueller, Elisabeth Epstein, Joseph W. Carlson

**Affiliations:** 1 Department of Obstetrics and Gynaecology, University Hospital of Bern and University of Bern, Bern, Switzerland; 2 Department of Oncology-Pathology, Karolinska Institutet, and Department of Pathology and Cytology, Karolinska University Hospital, Stockholm, Sweden; 3 Institute of Pathology, University Bern, Bern, Switzerland; 4 Department of Clinical Science and Education Karolinska Institutet, and Department of Obstetrics and Gynaecology Södersjukhuset, Stockholm, Sweden; Ohio State University Wexner Medical Center, UNITED STATES

## Abstract

**Background and purpose:**

Individualized therapy in endometrial cancer, the most common gynaecologic cancer in the developed world, focuses on identifying specific molecular subtypes. Mutations in the exonuclease domain of the DNA polymerase epsilon (POLE) gene define one such subtype, which causes an ultra-mutated tumour phenotype. These tumours may have an improved progression-free survival and may be receptive to specific therapies. However, the clinical phenotype of these tumours is unknown. The objective of this study was to evaluate the clinical and genetic features of POLE-mutated tumours from a large cohort of women whose cases are characterized by: (1) the availability of detailed clinical and lifestyle data; (2) mutation analysis; and (3) long-term follow-up.

**Methods:**

A total of 604 patients with endometrial cancer were included in the study. Data from a detailed questionnaire, including lifestyle and family history information, provided extensive pertinent information on the patients. Sequencing of exons 9–14 of the POLE gene was performed. Follow-up data were gathered and analysed.

**Results:**

Hotspot pathogenic POLE mutations were identified in N = 38/599 patients (6.3%). Patients with a POLE-mutated tumour were significantly younger, were more often nulliparous, and had a history of smoking. POLE-mutated tumours were more frequently aneuploid. Prognosis for patients with hotspot POLE-mutated tumours was significantly better in comparison with patients with non-mutated tumours; however careful selection of pathogenic mutations is essential to the definition of this prognostically favourable group.

**Conclusions:**

This study demonstrates that POLE-mutated endometrial cancer is significantly associated with previously unknown clinicopathologic characteristics. Outcome in POLE-mutated tumours was excellent in cases with hotspot mutations. Our results suggest that prediction of excellent outcome in cases of POLE-mutated EMCA should be restricted to cases of EMCA with hotspot mutations until further data are available on the rising number of mutations with unknown significance.

## Introduction

Mutations in the exonuclease domain of the polymerase epsilon (POLE) gene are associated with an ultra-mutated genetic phenotype, increased neoantigen load, increased tumour infiltrating lymphocytes, and potential responsiveness to immune therapy [1–5]. POLE mutations have been identified in 7–12% of endometrial cancers (EMCA), as well as in 1–2% of colorectal cancers, and have been described in rare cases of breast, pancreatic, stomach, lung, ovarian, and brain tumours [6]. These tumours show a better progression-free survival when compared to other tumours of similar type, grade, and stage [7–11]. The importance of the POLE gene in DNA replication was initially described in 2007 in studies on yeast, which showed its function in removing errors during leading-strand replication [12]. Mutations in this gene were first described in colorectal cancer in 2012, and its importance has been described recently in EMCA [13,14]. The defect in the exonuclease proofreading domain leads to an extremely high mutation load [5,15]. This, in turn, appears to lead to increased neoantigen production and activation of the patient’s immune system. This association with increased tumour-infiltrating lymphocytes, mostly with CD8 T-cells, may explain why these tumours appear to have an improved prognosis [1,4,16–18].

POLE mutations define a specific molecular subgroup of EMCA, with both therapeutic and prognostic significance. Ultra-mutated tumours with POLE mutations were identified as one of the four proposed molecular subgroups in a publication of The Cancer Genome Atlas (TCGA) based on their integrated genomic, transcriptomic, and proteomic characterization of 373 cases of EMCA [19].

The clinical characteristics of patients whose tumours have a POLE mutation are not clear. Several studies of EMCA cohorts have indicated that patients with POLE-mutated tumours are younger. A large retrospective study in colorectal cancer demonstrated that POLE-mutated tumours occur in younger patients more often than non-POLE-mutated tumours (median 54.5 years for POLE-mutated tumours vs. 67.2 years for non-POLE-mutated tumours). In that particular study, tumours were identified primarily in men. Previous studies have also indicated that women with POLE-mutated EMCA tend to have lower BMI. POLE-mutated tumours are described as having excellent outcome, even in high-grade endometrioid histology, usually known to be associated with a more aggressive tumour. Therefore, it has been proposed that POLE-mutated tumours might need less aggressive treatment and, if they do need treatment, they might be eligible for checkpoint inhibitors, due to the highly immunogenic character of these tumours [3,20]. The role of other clinical factors of potential importance in EMCA is unclear. In addition, the definition of pathogenic mutations used to classify a tumour as “POLE mutated” is not yet clearly defined.

The goal of this study was to evaluate the clinical and genetic features of POLE-mutated tumours from a large cohort of women whose cases are characterized by: (1) the availability of detailed clinical and lifestyle data; (2) mutation analysis; and (3) long-term follow up.

## Materials and methods

### Cohort description

The combined Karolinska-Bern cohort (KImBer, N = 604) includes patients diagnosed at the Karolinska University Hospital (KS) and the University Hospital Bern (Bern). The KS patients were enrolled prospectively in the International Endometrial Tumour Analysis (IETA) study conducted between February 2011 and January 2016 for expert ultrasound assessment, after the diagnosis of EMCA through biopsy, D&C, or hysteroscopic resection [21]. Median follow-up period was 34 months (0–75 months). The cohort reflects the Stockholm population inasmuch as all patients with EMCA in the Stockholm region are treated at the KS. Responses to an extensive patient questionnaire were obtained at the time of diagnosis. The Bern cohort was gathered retrospectively, selected by the availability of tumour tissue after surgery from patients with a diagnosis of EMCA who had consented between 2004 and 2015 to the use of their tissue for research. The University Hospital of Bern is a tertiary referral clinical; the patients in the cohort are therefore representative of high-risk tumours and/or high-risk patients. Median follow-up time was 59.6 months (0–156 months). DNA isolation and tissue microarray construction were the same for both cohorts.

### Clinical and mutation analysis data

For the reporting of all methods and results, the REMARK guidelines were applied [22,23]. The clinical data, including preoperative patient characteristics, therapies, histology data, and follow-up, were obtained from the respective hospital internal electronic databases. Additionally, patients in the KS cohort were evaluated via a more detailed questionnaire, filled out in discussion with one of the co-authors (EE). These parameters included information on personal cancer history (subdivided by type: ovarian cancer, breast cancer, colon cancer, and other cancer), family history of cancer (ovarian cancer, breast cancer, colon cancer, and other cancer), lifestyle (smoking, alcohol consumption, and exercise), body measurements (height, weight, waist measurement, and bra cup size), and menstrual history. Risk classification was applied following the ESMO-ESGO-ESTRO consensus [24].

For survival status, patients were categorized as “alive,” “death due to disease,” “death due to other cause,” or “death of unknown cause.” Overall survival (OS) was calculated from date of surgery until death from any cause or until the date of last follow-up. Disease-specific survival (DSS) was the time from surgery to death due to disease. Progression-free survival (PFS) was the time from surgery to recurrence or progression (based on clinical evidence or diagnosis from imaging or biopsy).

The histopathological diagnoses were all reviewed by two experienced gynaecological pathologists (JC and TR), and diagnoses were made as defined in the WHO 2014 classification of EMCA. The following parameters were extracted from the pathology reports: microcystic, elongated, and fragmented (MELF) patterns, present or not; depth of invasion; cervical invasion; lymph vascular space invasion (LVSI); and ploidy.

For the identification of POLE mutations, genomic DNA was isolated from punches from formalin-fixed paraffin-embedded tumour tissue after choosing a region with >60% tumour and <20% necrosis. After quality control and purification (S1 File), bidirectional Sanger sequencing was performed according to standard protocols, using M13-tailed primers on an ABI 3500 Genetic Analyzer (Life Technologies, Carlsbad, CA, USA) and a BigDye Terminator v3.1 Cycle Sequencing Kit (Life Technologies). Mutation analysis of exons 9–14 (in the Bern cohort 9, 12, 13, and 14) was done using Mutation Surveyor software (SoftGenetics, State College, PA, USA) as well as manual inspection. Non-synonymous mutations were confirmed by resequencing and, in the cases of mutations with unknown predicted pathogenicity, DNA from normal tissue (for example, myometrium) was extracted and sequenced for the described mutation in order to exclude germline variants. After confirming that mutations were somatic, the pathogenic impact of the mutation was annotated in three groups: hotspot mutation (P286R, V411L, S297F, A456P and S459F), POLE mutation with published high total mutational burden (TMB) (TMB>100Mb) (in our cohort A465V, D462Y, P436H), POLE mutation of unknown significance (VUS). For the subsequent analysis, except where specifically stated, only the POLE hotspot mutations were defined as POLE mutated.

Research ethics approval was obtained by the Regional Ethical Review Board of Stockholm, (reference number: 2011/34) and the Ethics Committee Bern, Switzerland (reference number: 2018–00479).

### Statistical analysis

Demographic and clinical-pathologic characteristics are presented using basic descriptive statistics. To further compare the characteristics of the two groups (POLE mutated and non-POLE mutated), Fisher’s exact test and independent t-tests were used. For variables not meeting the assumptions of the t-test equivalent, a non-parametric test was used. Survival analysis was performed by using Kaplan-Meier curves and with log rank test. For assessing risk factors for recurrence (PFS) and for DSS, Cox regression analysis was performed to evaluate the effect of all the different parameters (POLE mutation, histology, age, Grade, FIGO, etc) on outcome. All analyses were performed using SPSS version 25.0 (IBM). All p values tests were two sided, and p values <0.05 were considered statistically significant.

## Results

### Patient characteristics

A total of 604 patients were included in the study: 349 from the KS cohort and 255 from the Bern cohort. In five patients, the DNA quality was insufficient for analysis of all exons, and these patients were therefore excluded. Sanger sequencing was successful in all tumours. A hotspot POLE mutation was identified in 38/599 patients (6.3%). Standard patient characteristics, available for all 599 patients, are presented in Table 1. Patients with POLE-mutated tumours were significantly younger at the time of diagnosis than patients with non-POLE-mutated tumours (60.1 vs 66.5 years; p = 0.000). Additionally, these patients were more often nulliparous (39.5% vs 22.6%; p = 0.028) and had a tendency towards lower BMI, although this difference was not statistically significant. Detailed patient characteristics and life-style information were available for most of the KS cohort (N = 342 out of a total of 349). The significant data are summarized in Table 2. The data show that patients with POLE-mutated tumours were significantly more often smokers or ex-smokers (p = 0.041). A non-significant trend was seen in the use of hormone replacement therapy (more frequent in non-POLE-mutated cases) and, interestingly, in cases with a family history of colon cancer. Four out of the five patients with a POLE-mutated EMCA and a family history of colon cancer showed a P286R mutation. None had a personal history of colon cancer and none showed micro-satellite instability.

**Table 1:** Clinicopathological characteristics.

	POLE mutated	No POLE mutation	p-value
	N = 38	N = 561	
**Age at time of diagnosis (mean, range)**	60,1 (41–80)	66,5 (31–93)	**0,000**
**BMI (mean, range)**	27,4 (20,4–41,5)	29,8 (16,4–58,6)	0,071
**Parity**			
** Nullipara**	15 (39,5%)	126 (22,5%)	
** Multipara**	23 (60,5%)	434 (77,5%)	**0,028**
**Menopausal status**			
** premenopausal**	6 (15,8%)	48 (8,6%)	
** Postmenopausal**	32 (84,2%)	513 (91,4%)	0,140
**Histology**			
** Endometrioid**	31 (81,6%)	468 (83,4%)	
** Non-endometrioid**	7 (18,4%)	93 (16,6%)	0,822
**Grade**			
** 1,2**	22 (57,9, 2%)	411 (73,3%)	
** 3**	16 (57,9%)	150 (26,7%)	0,059
**FIGO**			
** I**	33 (86,8,2%)	414 (73,8%)	
** II**	2 (5,3%)	53 (9,4%)	
** III**	3 (7,9%)	67 (11,9%)	
** IV**	0 (0%)	27 (4,8%)	0,282
**Lymphadenectomy**			
** LN positive**	2 (8,7%)	61 (22%)	
** LN negative**	21 (91,3%)	216 (78.0%)	0,183
** No LND performed**	16 (42,1%)	310 (55,6%)	0,130
**Tumour size mm**	32,06	34,03	0,482
**Cervical invasion**			
** None**	35 (92,1)	440 (78,6%)	
** Mucosal**	0 (0%)	29 (5,2%)	
** Stromal**	3 (7,9%)	91 (16,3%)	0,113
**Depth of invasion**			
** Intramucosal**	1 (2,6%)	52 (9,3%)	
** <50%**	23 (60,5%)	286 (51,1%)	
** >50%**	14 (36,8%)	222 (39,6%)	0,292
**LVSI**			
** Negative**	23 (60,5%)	414 (73,8%)	
** Positive**	15 (39,5%)	147 (26,2%)	0,075
**Ploidity**			
** Aneuploid**	13 (48,1%)	90 (29,6%)	
** Diploid**	14 (51,9%)	214 (70,4%)	**0,046**
**ESMO Group**			
**Low risk**	14 (36,8%)	224 (39,9%)	
**Intermediate risk**	1 (11,7%)	69 (12,3%)	
**High intermediate risk**	9 (23,7%)	49 (8,7%)	
**High risk**	14 (36,8%)	189 (33,7%)	
**Advanced/metastatic**	0 (0%)	30 (5,3%)	**0,010**

Missing data: Parity N = 1, BMI N = 107, depth on invasion: N = 1, Tumour size: N = 55; cervical invasion = 1, Ploidy only KS cohort (Data on 331 patients). Statistical analysis: numerical independent t-test, categorical Fisher’s exact

**Table 2:** Detailed patient characteristics of POLE mutated tumours.

	POLE mutated	Non-POLE mutated (N = 306)	p-value
	(N = 36)		
**Smoking**			
**Never**	17	259	
**Ever or currently**	11	55	**p = 0,018**
**Family history of EMCA**			
**(Number of relatives)**			
** 0**	25 (89,3%)	285 (89,3%)	
** 1**	2 (7,1%)	25 (8,0%)	
** 2**	1 (3,6%)	3 (1%)	
** 3**	0	1 (0,3%)	p = 0,655
**Family History of colorectal cancer**			
**(Number of relatives)**			
** 0**	23 (82,1%)	294 (93,6%)	
** 1**	5 (17,9%)	17 (5,4%)	
** 2**	0	2 (0,6%)	
** 3**	0	1 (0,3%)	p = 0,078
**HRT yes**	7 (25,0%)	77 (24,5%)	p = 0,955
**Bra cup**			
** Small (0–3)**	21	198	
** Large (4–8)**	8	116	p = 0,162
**Waist length (mean)**	98,04cm	99,01	p = 0,161
**Height (mean)**	165,0cm	165, 0	p = 0,995

Data on 342 patients (most of the KS cohort, 7 missing data on IETA questionnaire). HRT: Hormone replacement therapy. Statistical analysis: numerical independent t-test, categorical Fisher’s exact

### Tumour histopathology

Histopathologic characteristics of the tumours are presented in Table 1. The POLE-mutated tumours were mostly, but not exclusively, endometrioid and low risk. No significant differences between the two groups were found with regard to the histologic type or tumour stage; the tumours have no clear defining histological phenotype. In numbers, there are more FIGO Stage I (86.8% vs 73.8%) and Grade 3 tumours (57.9% vs 26.7%) but these findings were not significant. In the POLE-mutated group, the non-endometrioid histologic types were four mixed carcinomas, one serous carcinoma, and two clear-cell carcinomas. There were significant differences among risk-group categories, notably with fewer advanced risk cases in the POLE mutated group. Data on ploidy of the tumours were available for the KS cohort only; for this cohort, the POLE-mutated tumours were significantly more often aneuploid.

### POLE mutations

In the KS cohort, where exons 9–14 were sequenced, no POLE mutations were found on exon 10. Only one POLE mutation was found on exon 11, which was classified as a non-pathogenic variant (ie VUS, not an ultramutated phenotype). Therefore, in the Bern cohort, only exons 9, 12, 13, and 14 were sequenced. The number of mutations found on each exon were: 31 on exon 9; 1 on exon 12; 13 on exon 13; and 11 on exon 14.

Thirty-eight tumours had at least one hotspot POLE mutation (see Table 3). An additional 19 tumours had mutations that were classified as not defined as hotspot mutations for the purposes of this study but have been previously described as having an ultramutated phenotype (N = 3). Finally, a number of variants of unknown significance were identified: “variants of unclear significance” with high pathogenic FATHMM predictive scores (N = 8), “variants of unclear significance” without any available FATHMM predictive score (N *=* 7), or non-pathogenic FATHMM score (N = 1). A subgroup analysis was performed looking at alternative definitions of POLE mutated EMCA. In this analysis, defining POLE mutated EMCA as only including hotspot mutations provided the best patient outcome. Including both hotspot and ultramutated tumors also showed an good outcome, and the survival benefit was significant (S2 File). Including hotspot, ultramutated, and “VUS with a pathogenic FATHMM score” in the POLE EMCA resulted in no significant survival difference between the groups (S2 File). For this purposes of all further discussion, POLE mutated EMCA included only hotspot mutations. All other cases were thus excluded and placed into the non-POLE mutated group.

**Table 3:** POLE mutations and classification.

Nucleotide substitution	Amino acid chang	Exon	Nr of mutations found	Decision pathogenic	FATHMM prediction score	Hyper-mutated phenotype described
**Hotspot mutations**						
**c.857C>G**	P286R	9	22	hotspot	1	yes
**c.1231G>C**	V411L	13	9	hotspot	0.99	yes
**c.1376C>T**	S459F	14	4	pathogenic	0.99	yes
**c.1366G>C**	A456P	14	1	pathogenic	0.99	yes
**c.890C>T**	S297F	10	1	pathogenic	0.99	yes
**Ultramutated**						
**c.1394C>T**	A465V	14	1	Pathol. phenotype	1	yes
**c.1384G>T**	D462Y	14	1	Pathol. phenotype		yes: 278.4
**c.1307C>A**	P436H	13	1	Pathol. phenotype		yes, 541.36
**Unclear significance with high prediction score**						
**c.1231G>A**	V411M	13	3	VUS	0.99	
**c.1370C>T**	T457M	14	2	VUS	1	
**c.808G>A**	V270M	9	1	VUS	0.99	
**c.1175A>G**	D392G	12	1	VUS	0.99	
**c.885G>A**	M295I	9	1	VUS	0.99	
**c.901G>A**	D301N	9	1	VUS	0.99	
**c.1283C>T**	A428V	13	1	VUS	0.99	
**c.1439C>T**	A480V	14	1	VUS	0.99	
**Unclear significance**						
**c.1240G>A**	D414N	13	1	VUS		
**c.1423C>T**	H475Y	14	1	VUS		
**c.887T>G**	I296S	9	1	VUS		
**c.1461G>A**	M487I	14	1	VUS		
**c.1190A>G**	Y397C	12	1	VUS		
**c.1103A>T**	D368V	11	1	VUS		
**c.844C>T**	P282S	9	1	VUS		
**Benign**						
**c.1371G>A**	T457T	14	1	benign	neutral score 0.04	

FATHMM = Functional Analysis through Hidden Markov Models; VSCS = Variant of strong clinical significance (pathogenic); VUS = Variant of unknown significance (non-pathogenic)

Table 3 shows the mutations found and the classification as hotspot, ultramutated, or VUS.

### Clinical outcomes

As described previously, only hotspot mutations were included in the POLE mutation group unless specifically mentioned. The non-POLE group includes all remaining cases unless otherwise specified. In the outcome analysis, 1/38 (2.7%) patient with POLE mutations showed recurrence, as compared to 89/526 (16.9%) in the non-POLE-mutated group (p = 0.023). One patient with a hotspot POLE-mutated tumour lacked follow-up data and was excluded from the recurrence analysis. Time to recurrence was 25 months for the patient with POLE mutation and a mean of 18.1 months (SD 18.8) for the non-POLE-mutated group (p = 0.745). The patient with recurrence had a serous histology with LVSI positive tumour, FIGO Stage 1, and had an adjuvant chemotherapy. In the POLE-mutated group, 37 (97.4%) were alive at last follow-up, and 1 (2.6%) died of disease. In the non-POLE-mutated group, 454 (81.7%) were alive, 56 (10.1%) died of disease, 15 (2.7%) died of unknown causes, 28 (5.0%) died of other causes, and 3 (0.5%) died due to treatment (p = 0.181). Five patients were lost to follow-up. Overall survival was 22.8 months (SD 14.09) and 26.2 months (SD 18.51) respectively (p = 0.645). Mean follow-up time was 47.5 months (12–155) vs 46.8 months (0–156) (p = 0.710).

**Fig 1:**
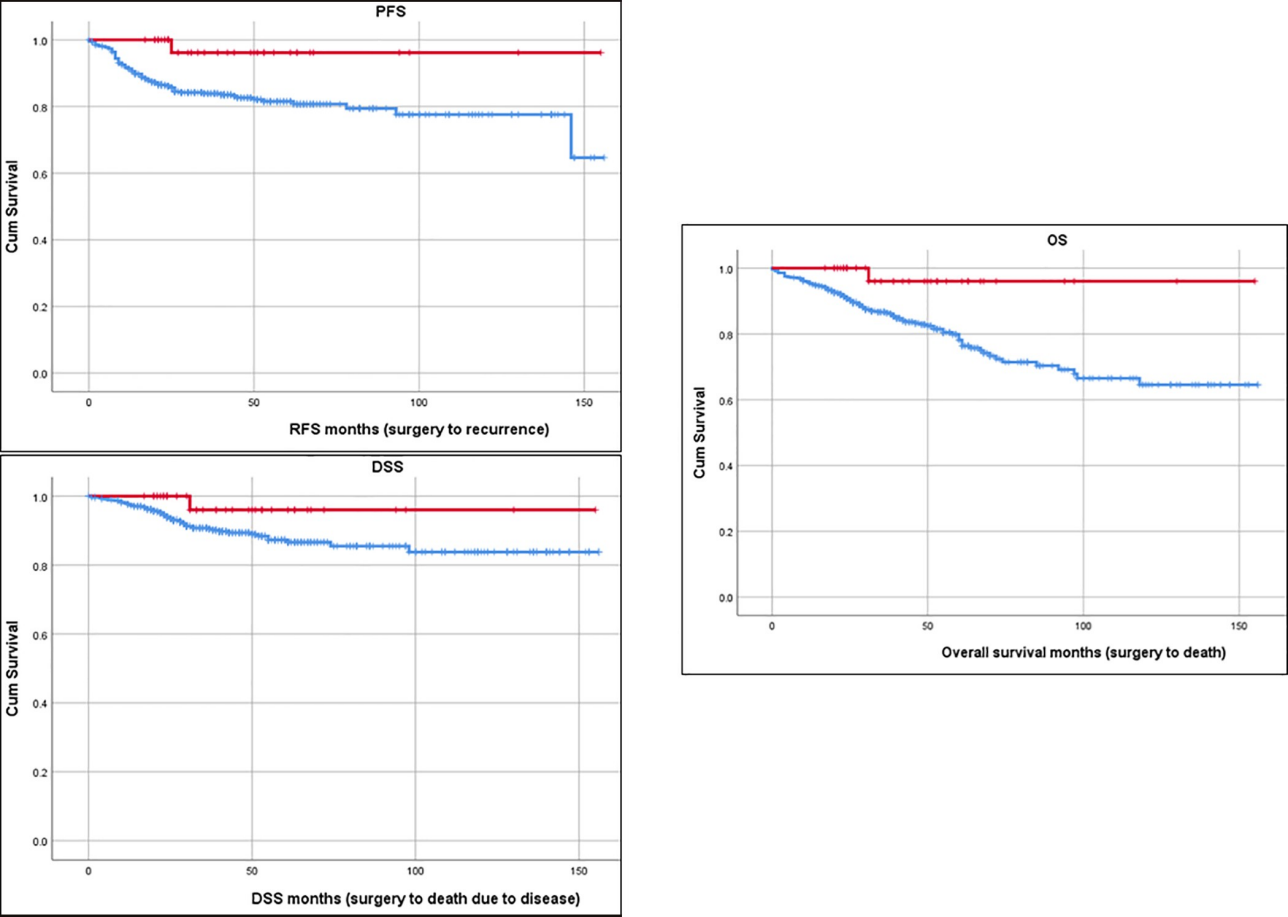
Kaplan Meier analysis of POLE vs. non POLE-mutated tumours for PFS, DSS and OS. PFS: Progression free survival (operation to recurrence or progression). DSS: Disease specific survival (operation to death due to disease). OS: Overall survival (operation to death of any cause). Red line: patients with POLE-mutated tumour, censored. Blue line: patients with no POLE-mutated tumour, censored.

Comparison of survival of both groups is shown in Fig 1, with the Kaplan-Meier curves for PFS, DSS, and OS. Patients with POLE-mutated tumours had a significantly better PFS and OS (og-rank results of 0.025, 0.147, and 0.023 respectively). Furthermore, applying Cox regression for analysing the risk of POLE mutations shows a hazard ratio for recurrence (PFS) of 0.145 (CI 0.020–1.043, p = 0.055) and for DSS of 0.258 (CI 0.036–1.862, p = 0.179), both being just non-significant.

In summary, the POLE-mutated tumour cases had a better outcome compared to the rest of the cohort.

Analysis of the surgical and adjuvant treatments was performed in order to observe the effect of treatment: hysterectomy (HE) with bilateral salpingo-oophorectomy (BSO) alone was performed in 16 (42.1%) versus 310 (55.4%) cases; HE/BSO/pelvic lymphadenectomy (PLND) was performed in 5 (13.2%) versus 85 (15.2%); and HE/BSO/PLND/Para-aortal lymphadenectomy (PALND) was performed in 17 (44.7%) vs 154 (27.5%) cases. In the non-POLE-mutated group, 11 (2%) underwent other types of surgery (including intestinal surgery); information was missing for one patient. In the POLE-mutated group, more LND were performed (57.9% vs 44.4%); this finding was not significant (p = 0.107). Both clinics are dedicated to minimally invasive surgery for EMCA; therefore, the surgery performed was minimally invasive (laparoscopy or robotic surgery) in 77.4% vs 81.5% (p = 0.464) cases. Of the 273 patients having at least a PLND, the mean number of lymph nodes removed was 32.9 vs 35.3 (p = 0.502); this shows that when a lymphadenectomy was performed, it was the same procedure in both groups and sufficiently extensive.

Concerning adjuvant treatment, 22 (57.9%) vs 323 (58.9%) (p = 0.899) had no treatment at all. Comparison of the different forms of adjuvant treatment showed that no significant difference exists between the groups (p = 0.783): (4 (10.5%) vs 37 (6.8%) had chemotherapy, 9 (23.7%) vs 99 (18.1%) had combined radio-chemotherapy, and 3 (7.9%) vs 89 (16.1%) had radiation alone (brachytherapy and/or pelvic radiotherapy); one patient in the POLE non-mutated group had hormonal treatment.

Subgroup analysis included endometrioid tumours (n = 465, POLE mutations in 30 cases) and Grade 3 endometrioid tumours (n = 72, POLE mutations in 10 cases). No recurrence was noted in the endometrioid group with POLE mutation; however, in the Cox-regression analysis for risk of recurrence, no significance was reached (p = 0.172; CI 0.001–3.884). In addition, analysis of the non-endometrioid tumours (N = 98) showed that the POLE mutation (N = 7) did not have a significant positive effect on survival.

In a further subgroup analysis of the effect of POLE mutation on outcome in EMCA, we performed Cox regression analysis on ESMO risk groups applied to our cohort [24]. Within each risk group, no significant difference could be seen as to whether or not a POLE mutation was present.

## Discussion

This study presents the phenotypic and mutational spectrum of a large cohort of POLE-mutated tumours. By combining extensive clinical data, including detailed information on lifestyle, POLE mutations, and survival outcome, this work identifies a few distinct phenotypic characteristics of women with POLE-mutated EMCA. First, women with POLE-mutated tumours are younger. Previous studies, including Bosse et al., already also showed a trend towards a lower age [10]. Differences in age of onset may be related to differences in cancer development. Indeed, the mean age of the diagnosis of simple hyperplasia is 50–54 and the mean age of the diagnosis of atypical hyperplasia is 60–64 [25]. In our cohort, the mean age of POLE-mutated tumours was 60.1 years versus 66.5 years in non-POLE mutated tumours. The existence of a POLE mutation makes a tumour susceptible to acquiring additional mutations and may accelerate the transition from precancerous lesion to cancer. The fully developed adenocarcinoma, however, may then be kept under control by high neoantigen loads and number of tumour-infiltrating lymphocytes, thus inducing a local immune response and explaining the good outcome [16,18,26]. A high BMI is known to be a risk factor for EMCA, favouring the usual type I EMCA [27]. Therefore, these findings might suggest a different pathogenic pathway than in the case of a typical type I EMCA.

In addition to the age factor and the trend towards lower BMI, patients with POLE-mutated tumours are more often current or former smokers. A recently published large study of the risk of smoking for cancer as well as a meta-analysis published in 2008 suggest that smoking may reduce the general risk of developing EMCA [28–30]. However, in premenopausal women, smoking was a secondary risk factor for developing EMCA in 5 out of 9 studies summarized in a meta-analysis published in 2016 [27]. Therefore, the identification of more POLE tumours in ever-smokers is intriguing. Smoking appears to cause mutations via direct action of carcinogens in smoke and smoke metabolites on DNA, but also via additional pathways, such as defects in DNA repair, as in the case of occasional POLE mutations [31]. A recent article demonstrates that clustered mutation signatures, combined with error-prone DNA repair, leads to accumulation of mutations in active, more important regions of the genome [32]. The degree to which these processes are active in EMCA needs to be evaluated in future studies.

Another interesting and important finding from the analysis of the histopathology of the tumours is that there is no significant difference between the histologic type or stage of the POLE-mutated versus the non-POLE tumours. In our cohort, POLE-mutated tumours do proceed to lymph-node metastasis and may also be non-endometrioid. In the sub-analysis of endometrioid grade 3 tumours (N = 72), there are significant differences in prevalence between the groups (23.7% vs 11.2% p = 0.022). This confirms the results from the other published large cohorts showing that POLE mutated tumours are more often endometrioid and high grade [10,14,33,34]. This finding is not new, but is an important confirmation, since several of these cohorts have had differences in the inclusion criteria. For example, the PORTEC studies included primarily intermediate to intermediate-high risk cases and the TCGA included primarily high-risk cases. The KImBer cohort represents a population with EMCA without pre-selection, therefore very representative of the general population.

From the KS cohort, information on ploidy shows that POLE-mutated tumours are significantly more often aneuploid than non-POLE-mutated tumours. Ploidy alone is known as a risk factor for more aggressive tumour biology. Furthermore, in these 331 patients, progression-free survival was significantly worse in the aneuploid group (log rank 0.000); this finding is consistent with the findings of other large studies on ploidy in EMCA [35]. Importantly, the role of ploidy is subtype specific. The POLE-mutated tumours were significantly more often aneuploid, but there was no difference in outcome between aneuploid and diploid tumours within the POLE-mutated group. This finding within a larger cohort confirms the results by Hoang et al, who examined the ploidy of different molecular subgroups; they also found that within the total cohort, aneuploid tumours had a worse outcome, but that within the POLE tumours there was no difference in PFS [36]. Usually, non-diploid cells are a sign of genomic instability, which is in general a risk for more aggressive tumour biology. The genomic instability in the POLE-mutated tumours is not due to aneuploidy but rather to the missing controls in DNA replication and therefore reflects a different mechanism; this may be the reason for the missing effect of ploidy on prognostic significance [37].

In analysis of the PFS and DSS, the hotspot POLE-mutated tumours do have a significantly better outcome; this finding confirms previously published data that POLE mutated tumours have a good outcome. Cohorts have been published from Vancouver, subgroups of PORTEC-1 and 2, the TCGA, Ohio, Calgary, Singapore, and combined PORTEC-NGO, presenting a total of over 150 POLE-mutated tumours with good to excellent survival rates [10,16,19,34,38–40]. Note, however, that many of these studies have included a range of POLE mutations in the “POLE mutated” group (summarized in Table 4)

**Table 4:** Mutations defined as pathogenic in previous cohorts.

Publication	Nr of POLE mutated tumors	Sequencing method	Mutations included in the “POLE mutated” group	Definition of pathogenic mutation
**Tomlinson et al 2013**	14	Sanger sequencing (codons 268–471)	P286R, S297P, V411L, A456P, A275V	Predictive and functional analysis
**McAlpine et al 2015**	39	Sanger sequencing exons 9 to 14	P286R, S297P, V411L, A456P, M295R, F367S/C, P436R, L424P, P441L, F367L, E396G	
**Ngeow et al 2016**	12	Nextgeneration sequencing and sanger sequencing to confirm	P286R, V411L, A456P, S459F A465F, M444K, S459P	In silico testing of mutations to define pathogenity
**Billingsley et al 2015 and Goodfellow et al 2017**	40/39	Sanger sequencing (residues 268–471)	P286R, S297P, V411L, A456P, P436R, A465F, A426V	assessed using mutation assessment prediction programs
**Bosse et al 2015**	63	Sanger sequencing exon 9 and 13 (PORTEC)	P286R, S297P, V411L, M299V, S297T	defined as pathogenic POLE proofreading mutations as variants absent from public germline sequence databases and previously confirmed as somatic variants associated with tumor ultramutation
**Köbel et al 2014**	8	Sanger sequencing exon 9–13	P286R, V411L, T278M, S297P	all mutation positive samples

References [10,40–44]

Sequencing of exons 9–14 of the POLE gene, as performed in this study, but also performed in numerous previous studies, has led to the identification of numerous non-synonymous mutations (summarized in Table 3). Interpretation of these mutations, and determination of their being “pathogenic” or not, is a growing issue that has been handled differently by different authors (Table 4). The relationship between a particular mutation and the clinical phenotype of “good prognosis” is not entirely clear. In this study, we defined a “POLE-mutated tumour” using a restrictive definition that included only five previously described hotspot mutations (P286R, V411L, S297F, A456P and S459F). Using this definition, POLE-mutated tumours showed the phenotypic findings we have described above. We also identified tumours with mutations that have been demonstrated to lead to a high TMB. A subgroup analysis including these in a “POLE-mutated” group also demonstrated a good prognosis. However, we found a large range of additional mutations where the significance is not clear and can pose difficulty in interpreting the results. By adding all the POLE mutations to the analysis, the outcome is not significantly better compared to no POLE mutation (Kaplan Meier curves presented in S2 File). Some of these mutations have been designated as “pathogenic” by other authors and tumours have been included in “POLE-mutated” groups based on them (see Table 4). This analysis shows that a correct selection of pathogenic mutations influences the results significantly and means that previous papers that have included mutations of unclear significance should perhaps be revisited. Our results suggest that the safe definition of POLE-mutated EMCA with prediction of an excellent outcome should, for the moment, be restricted to endometrioid EMCA with the five hotspot mutations we have listed, with perhaps the inclusion of the high TMB mutations. We would argue that no other mutations should be included in this group until their biology is more completely understood.

As seen in the work of Framton et al., in analysing a large number of somatic mutated tumours, the variance of the TMB is large and therefore shows that mutation alone does not have to result in a high TMB [4]. In addition to the characteristic of a high mutation rate, the POLE-mutated tumours as classified by the TCGA have specific nucleotide missense mutations such as TCT→TAT and TCG→TTG mutations [5,19]. Clearly, more research is needed to understand which POLE mutations lead to a phenotype with better clinical outcome and whether this is due to the POLE mutation alone leading to an ultramutation or to another mutation within the many mutations further down the road, leading to the good outcome.

In the KS cohort, no mutations were found in exons 10 and 11. This supports the recent literature [44], so it is probable that the sequencing of exons 9, 12, 13, and 14 is sufficient but also necessary for diagnosing pathogenic POLE mutations.

One limitation of this study is the lack of additional molecular characterization. Several studies have indicated that POLE-wildtype EMCA can have deficiencies in other molecular pathways, such as mismatch repair, and can even be p53 mutated. While the original TCGA paper and the ProMisE classifier include tumours with multiple classifiers (and have demonstrated the POLE group as having a distinctly favourable prognosis), other groups, notably the papers focusing on the PORTEC cohort, have excluded cases with multiple classifiers [10,45,46]. This question needs to be addressed in future studies.

## Conclusions

In this large representative cohort, patients with POLE-mutated EMCA are younger, more often nulliparous, and more often current or prior smokers. POLE mutations do lead to a better outcome; however, a careful definition of pathogenic POLE mutation is needed. Subgroup analysis of identified POLE mutations demonstrate that a restrictive definition of “POLE-mutated EMCA” is necessary to achieve the reported good survival, as including variants of uncertain significance leads to inclusion of cases with worse prognosis. Possibly, the identification of more factors, such as additional mutations or total mutational burden, might be needed to identify clearly pathogenic POLE mutations with the associated excellent prognosis. These results show that finding a POLE mutation alone has limits in identifying a clear clinical cohort and more research is needed to better understand the mechanisms of the different POLE mutations. In anticipating of the use of checkpoint inhibitors for ultramutated tumours, this molecular marker may evolve to be an important factor in treatment for EMCA, especially in patients with advanced or recurrent disease.

## Supporting information

S1 File Quality control and purification. (DOCX)

S2 File Kaplan Meier Curves for hotspot POLE mutations with hypermutated mutations classified as POLE mutated and for all POLE mutations (hotspot and hypermutated and VUS with high prediction score). Red line: defined POLE-mutated,
Blue line: no POLE mutation.
(DOCX)
