# Supplementary material for: Phenotype of POLE-mutated endometrial cancer
Source: PLoS One. 2019 Mar 27;14(3):e0214318. doi: 10.1371/journal.pone.0214318 (PMC6436745; doi:10.1371/journal.pone.0214318)
Supplement: S1 File — (DOCX) [file pone.0214318.s001.docx]

Supplementary Information Appendix S1

DNA extraction methods:

Chemagic DNA FFPE external lysis VD101124.che provided by PerkinElmer was used for purification. DNA quality control was performed with Illumina FFPE QC Kit.

Polymerase Chain Reaction (PCR) amplification was done using M13-tailed primers with PCR-primers. PCR products were purified using either a spin column commercial kit or ExoSap-IT (Thermo Fisher Scientific, Waltham, MA, USA). In the Swiss cohort the QS GeneRead™ DNA FFPE was used for DNA extraction.
