# Supplementary material for: Phenotype of POLE-mutated endometrial cancer
Source: PLoS One. 2019 Mar 27;14(3):e0214318. doi: 10.1371/journal.pone.0214318 (PMC6436745; doi:10.1371/journal.pone.0214318)
Supplement: S2 File — Red line: defined POLE-mutated, Blue line: no POLE mutation. (DOCX) [file pone.0214318.s002.docx]

Kaplan Meier Curves, variant definition of POLE group 1: “POLE mutated group” (red line) includes both tumors with hotspot mutations and tumors with mutations previously reported as ultramutated (mutations listed in Table 3). All other cases are in the “non-POLE mutated” group (blue line).


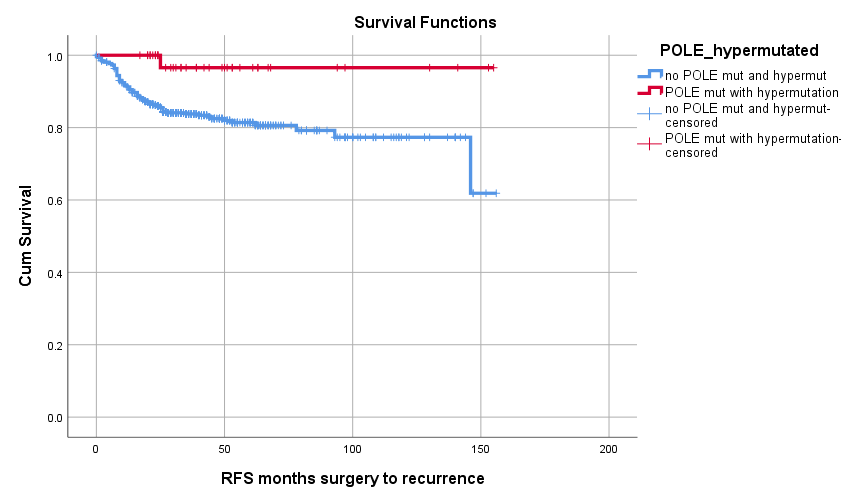
 Log rank 0.015


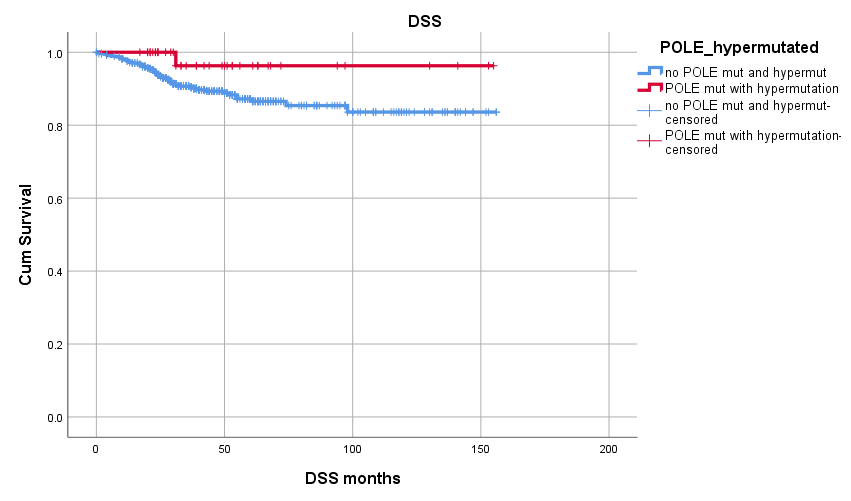
 Log rank 0.111


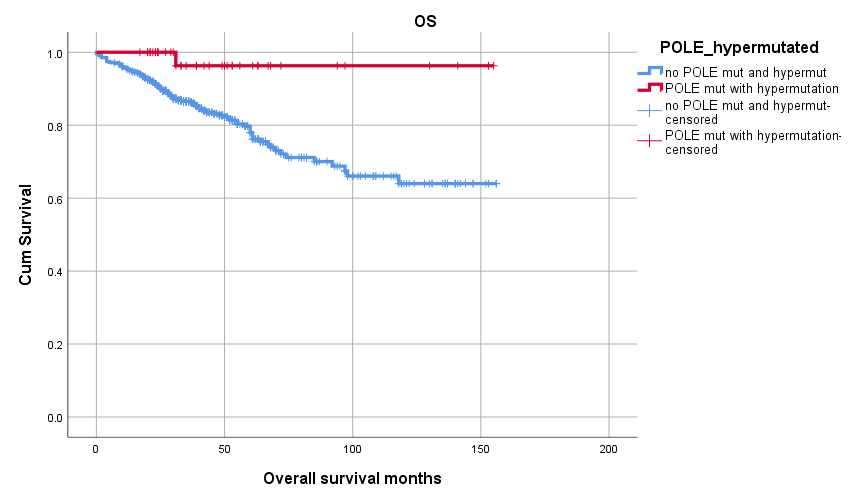
 Log rank p= 0.013

Kaplan Meier Curves, variant definition of POLE group 2. “POLE mutated group” (red line) includes tumors with hotspot mutations, mutations previously reported as ultramutated and unclear mutations with a high prediction score (mutations listed in Table 3). All other cases are in the “non-POLE mutated” group (blue line).


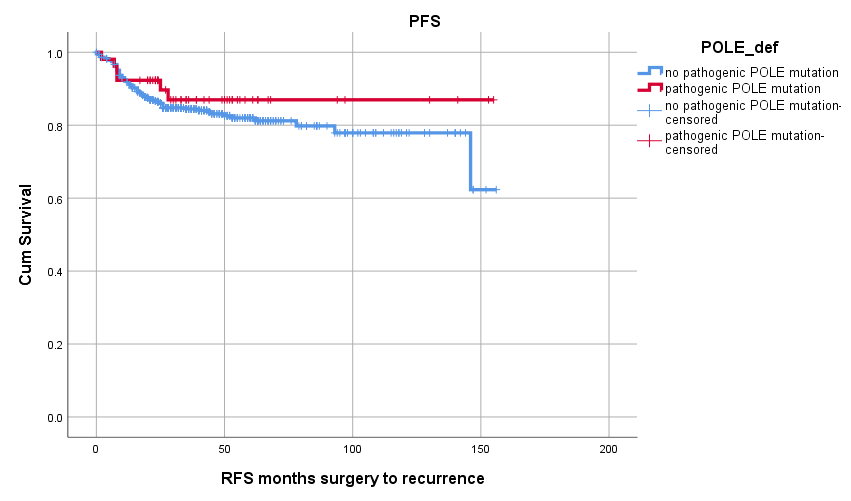


Log rank 0.329


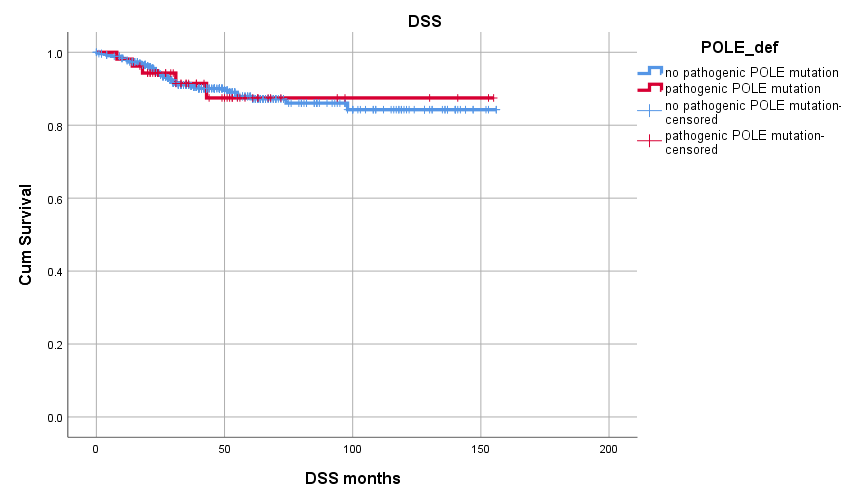
 Log rank 0.988


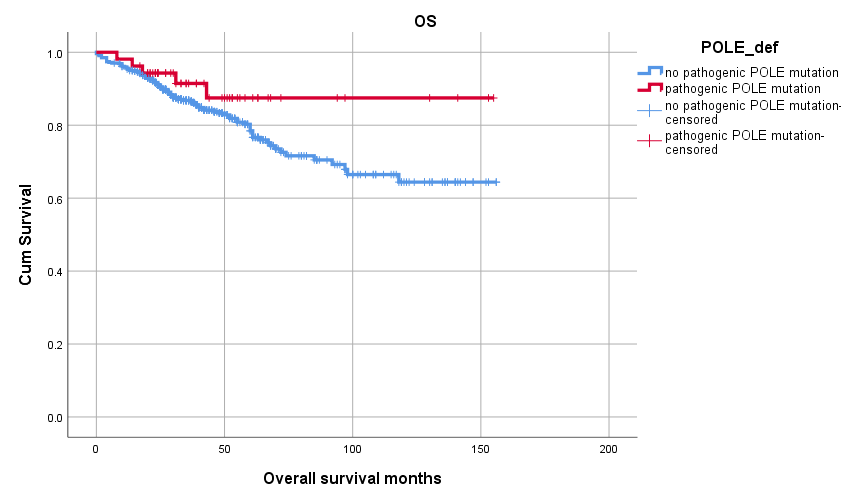
 Log rank p=0.154
